# The extent of functionality in the human genome

**DOI:** 10.1186/1877-6566-7-2

**Published:** 2013-07-15

**Authors:** John S Mattick, Marcel E Dinger

**Affiliations:** Garvan Institute of Medical Research, Darlinghurst, NSW 2010 Australia; St Vincent’s Clinical School, University of New South Wales, Kensington, NSW 2052 Australia; School of Biotechnology & Biomolecular Sciences, University of New South Wales, Kensington, NSW 2052 Australia

## Abstract

Recently articles have been published disputing the main finding of the ENCODE project that the majority of the human genome exhibits biochemical indices of function, based primarily on low sequence conservation and the existence of larger genomes in some ostensibly simpler organisms (the C-value enigma), indicating the likely presence of significant amounts of junk. Here we challenge these arguments, showing that conservation is a relative measure based on circular assumptions of the non-functionality of transposon-derived sequences and uncertain comparison sets, and that regulatory sequence evolution is subject to different and much more plastic structure-function constraints than protein-coding sequences, as well as positive selection for adaptive radiation. We also show that polyploidy accounts for the higher than expected genome sizes in some eukaryotes, compounded by variable levels of repetitive sequences of unknown significance. We argue that the extent of precise dynamic and differential cell- and tissue-specific transcription and splicing observed from the majority of the human genome is a more reliable indicator of genetic function than conservation, although the unexpectedly large amount of regulatory RNA presents a conceptual challenge to the traditional protein-centric view of human genetic programming. Finally, we suggest that resistance to these findings is further motivated in some quarters by the use of the dubious concept of junk DNA as evidence against intelligent design.

## Introduction

Recently there has been renewed discussion and controversy surrounding the extent and density of biochemical and biological functionality in the human genome (Graur et al. 2013; Doolittle 2013; Niu and Jiang 2013), prompted by the conclusion of the recent ENCODE studies (Dunham et al. 2012), following earlier analyses (Pheasant and Mattick 2007), that much if not most of the human genome may be functional. In particular the paper by Graur et al. 2013 has attracted particular attention because, unusually for a scientific paper, it employs not only logical argument to dispute but also sarcasm to ridicule the ENCODE conclusions.

## Review

Putting polemic and ideology (see below) aside for the moment, the substantive scientific argument of Graur et al. is based primarily on the apparent lack of sequence conservation of the vast majority (~90%) of the human genome, suggesting that this indicates lack of selective constraint (and therefore function). The fundamental flaw, however, in this argument is that conservation is relative, and its estimation in the human genome is largely based on the questionable proposition that transposable elements, which provide the major source of evolutionary plasticity and novelty (Brosius 1999), are largely non-functional. This argument also overlooks a number of other assumptions and considerations that are tacitly embedded in conservation comparisons and their interpretation (Pheasant and Mattick 2007):

(i) relative conservation imputes function, but lack of (discernable) conservation imputes nothing (Pang et al. 2006), especially when there may be high turnover (Smith et al. 2004; Frith et al. 2006), different evolutionary rate classes in different types of functional elements (Taylor et al. 2006; Oldmeadow et al. 2010), and/or extended evolutionary distances involved (think ‘*frere*’ and ‘*brother*’ for a linguistic analogy);
(ii) like words, regulatory sequences have more relaxed structure-function constraints than protein-coding sequences, which encode analog devices with strict chemical requirements. Indeed this is well supported by comparative analysis of gene promoters, which nobody disputes are functional, but where orthologous function can be retained over large evolutionary distances in the absence of any recognizable primary sequence conservation (see e.g. Fisher et al. 2006);
(iii) regulatory sequences are the main genetic substrates for the exploration of phenotypic diversity in animals, by orchestrating the differential expression of a relatively stable and largely orthologous set of protein-coding genes (Pheasant and Mattick 2007; Taft et al. 2007; Carroll 2008), which diverge under positive selection for lineage-specific adaptive radiation;
(iv) the conclusion of lack of conservation of most of the human genome is largely based on a circular comparison with the rate of evolution of pan-mammalian ancient ‘repeats’, a slightly pejorative term referring to transposon-derived sequences (many with RNA origins), which are assumed to be largely non-functional and therefore evolving ‘neutrally’. That is, one assumes that a subset of the genome is evolving neutrally and is therefore indicative of the rate of unconstrained divergence, then finds that most of the rest of the genome is behaving similarly, which is therefore concluded to also be non-functional. If the first assumption is incorrect, and increasing evidence suggests that it may be (Oldmeadow et al. 2010; Faulkner et al. 2009; Baillie et al. 2011) (although this is disputed in Graur et al. 2013), the derived conclusion of non-functionality of the rest of the genome is also incorrect (Pheasant and Mattick 2007).
  The fact is we simply do not know - most elements in the human genome have not been subject to functional analysis, which itself is fraught with ascertainment difficulties (see e.g. Lewejohann et al. (2004) for a retrotransposon-derived example). While others have provided superficially independent evidence that ancient repeats are neutrally evolving, based on indel distribution rather than primary sequence comparison (Lunter et al. 2006), this is subtly subject to similar circular logic and lack of acknowledgement that protein-coding (and some miRNA) sequences may have structure-function constraints and therefore mutational patterns different from those in cis-regulatory sequences and other classes of trans-acting regulatory RNAs that emanate from the genome (Pang et al. 2006; Dinger et al. 2009);
(v) even if ancient repeats are neutrally evolving (which we think unlikely), the extant comparison set is restricted to those whose orthology is recognizable, some barely so, and therefore represents the more conserved end of a starting population whose full original distribution is unknown, thereby underestimating to an unknown extent the true ‘neutral’ evolution rate and therefore the extent of conservation of the remainder of the genome (Pheasant and Mattick 2007).

The other substantive argument that bears on the issue, alluded to in the quotes that preface the Graur et al. article, and more explicitly discussed by Doolittle (Doolittle 2013), is the so-called ‘C-value enigma’ , which refers to the fact that some organisms (like some amoebae, onions, some arthropods, and amphibians) have much more DNA per cell than humans, but cannot possibly be more developmentally or cognitively complex, implying that eukaryotic genomes can and do carry varying amounts of unnecessary baggage. That may be so, but the extent of such baggage in humans is unknown. However, where data is available, these upward exceptions appear to be due to polyploidy and/or varying transposon loads (of uncertain biological relevance), rather than an absolute increase in genetic complexity (Taft et al. 2007). Moreover, there is a broadly consistent rise in the amount of non-protein-coding intergenic and intronic DNA with developmental complexity, a relationship that proves nothing but which suggests an association that can only be falsified by downward exceptions, of which there are none known (Taft et al. 2007; Liu et al., 2013).

In contrast to these uncertain indices, estimations and interpretations, the major fact to emerge from the ENCODE studies (Birney et al. 2007; Dunham et al. 2012) and their predecessors (Cheng et al. 2005; Carninci et al. 2005) is that the vast majority of the mammalian genome is differentially transcribed in precise cell-specific patterns (Mercer et al. 2008) to produce large numbers of intergenic, interlacing, antisense and intronic non-protein-coding RNAs, which show dynamic regulation in embryonal development (Dinger et al. 2008; Guttman et al. 2011; Ng et al. 2012), tissue differentiation (Sunwoo et al. 2009; Pang et al. 2009; Mercer et al. 2010; Askarian-Amiri et al. 2011) and disease (Gupta et al. 2010; Khaitan et al. 2011), with even regions superficially described as ‘gene deserts’ expressing specific transcripts in particular cells (Mercer et al. 2012; Roberts and Pachter 2011). Moreover, there is increasing evidence of their functional relevance (Mattick 2009b) and that a major function of these noncoding RNAs is to guide chromatin-modifying complexes to their sites of action, to supervise the epigenetic trajectories of development (Mattick and Gagen 2001; Dinger et al. 2008; Nagano et al. 2008; Pandey et al. 2008; Khalil et al. 2009; Mattick et al. 2009; Koziol and Rinn 2010; Spitale et al. 2011) - which appears to comprise a far greater fraction of human genetic programming than expected (Mattick 2004) in order to specify the architecture of the organism at a level of detail well beyond mere cell-type specification (Mattick et al. 2010).

Given these observations, we would submit that differential expression (including extensive alternative splicing) of RNAs is a far more accurate guide to the functional content of the human genome than logically circular assessments of sequence conservation, or lack thereof. Assertions that the observed transcription represents random noise (tacitly or explicitly justified by reference to stochastic (‘noisy’) firing of known, legitimate promoters in bacteria and yeast), is more opinion than fact and difficult to reconcile with the exquisite precision of differential cell- and tissue-specific transcription in human cells (for a recent debate see van Bakel et al. 2010; Clark et al. 2011). Moreover, where tested, these noncoding RNAs usually show evidence of biological function in different developmental and disease contexts, with, by our estimate, hundreds of validated cases already published and many more en route, which is a big enough subset to draw broader conclusions about the likely functionality of the rest. It is also consistent with the specific and dynamic epigenetic modifications across most of the genome, and concurs with the ENCODE conclusion that 80% of the genome shows biochemical indices of function (Dunham et al. 2012). Of course, if this is true, the long-standing protein-centric zeitgeist of gene structure and regulation in human development will have to be reassessed (Mattick 2004, 2007, 2011), which may be tacitly motivating the resistance in some quarters.

There may also be another factor motivating the Graur et al. and related articles (van Bakel et al. 2010; Scanlan 2012), which is suggested by the sources and selection of quotations used at the beginning of the article, as well as in the use of the phrase “evolution-free gospel” in its title (Graur et al. 2013): the argument of a largely non-functional genome is invoked by some evolutionary theorists in the debate against the proposition of intelligent design of life on earth, particularly with respect to the origin of humanity. In essence, the argument posits that the presence of non-protein-coding or so-called ‘junk DNA’ that comprises >90% of the human genome is evidence for the accumulation of evolutionary debris by blind Darwinian evolution, and argues against intelligent design, as an intelligent designer would presumably not fill the human genetic instruction set with meaningless information (Dawkins 1986; Collins 2006). This argument is threatened in the face of growing functional indices of noncoding regions of the genome, with the latter reciprocally used in support of the notion of intelligent design and to challenge the conception that natural selection accounts for the existence of complex organisms (Behe 2003; Wells 2011).

## Conclusions

It is our position that these arguments are misguided. Indeed, we have refuted the specific claims that most of the observed transcription across the human genome is random (Clark et al. 2011; Mercer et al. 2012) and put forward the case over many years that the appearance of a vast layer of RNA-based epigenetic regulation was a necessary prerequisite to the emergence of developmentally and cognitively advanced organisms (Mattick 1994; Mattick and Gagen 2001; Mattick 2004; Amaral et al. 2008; Mattick 2009a, 2011). This case is, moreover, entirely consistent with the broad tenets of evolution by natural selection, although it may not be easily reconcilable with current population theory and current ideas of evolutionary neutrality. In any case, that our understanding of the remarkably complex processes underlying the molecular evolution of life, including the likely evolution of evolvability (Mattick 2009c), is incomplete should not be surprising. With the emergence of transformative technologies, such as massively parallel sequencing, which provide tools to view the inner molecular workings of the genome that were inconceivable less than a decade ago, it is as important as ever that we as scientists remain open to observations that challenge even the most fundamental paradigms that exist within biology today.
